# Rotational Molding of Linear Low-Density Polyethylene Composites Filled with Wheat Bran

**DOI:** 10.3390/polym12051004

**Published:** 2020-04-26

**Authors:** Aleksander Hejna, Mateusz Barczewski, Jacek Andrzejewski, Paulina Kosmela, Adam Piasecki, Marek Szostak, Tairong Kuang

**Affiliations:** 1 Department of Polymer Technology, Gdańsk University of Technology, Narutowicza 11/12, 80-233 Gdańsk, Poland; paulina.kosmela@pg.edu.pl; 2 Institute of Materials Technology, Poznan University of Technology, Piotrowo 3, 61-138 Poznań, Poland; mateusz.barczewski@put.poznan.pl (M.B.); jacek.andrzejewski@put.poznan.pl (J.A.); marek.szostak@put.poznan.pl (M.S.); 3 Institute of Materials Engineering, Poznan University of Technology, Jana Pawła II 24, 60-965 Poznań, Poland; adam.piasecki@put.poznan.pl; 4 College of Material Science and Engineering, Zhejiang University of Technology, Hangzhou 310014, China; kuangtr@zjut.edu.cn

**Keywords:** rotational molding, polyethylene, wheat bran, waste management, wood polymer composites, recycling

## Abstract

Application of lignocellulosic fillers in the manufacturing of wood polymer composites (WPCs) is a very popular trend of research, however it is still rarely observed in the case of rotational molding. The present study aimed to analyze the impact of wheat bran content (from 2.5 wt.% to 20 wt.%) on the performance of rotationally-molded composites based on a linear low-density polyethylene (LLDPE) matrix. Microscopic structure (scanning electron microscopy), as well as physico-mechanical (density, porosity, tensile performance, hardness, rebound resilience, dynamic mechanical analysis), rheological (oscillatory rheometry) and thermo-mechanical (Vicat softening temperature) properties of composites were investigated. Incorporation of 2.5 wt.% and 5 wt.% of wheat bran did not cause significant deterioration of the mechanical performance of the material, despite the presence of ‘pin-holes’ at the surface. Values of tensile strength and rebound resilience were maintained at a very similar level, while hardness was slightly decreased, which was associated with the porosity of the structure. Higher loadings resulted in the deterioration of mechanical performance, which was also expressed by the noticeable rise of the adhesion factor. For lower loadings of filler did not affect the rheological properties. However, composites with 10wt.% and 20 wt.% also showed behavior suitable for rotational molding. The presented results indicate that the manufacturing of thin-walled products based on wood polymer composites via rotational molding should be considered a very interesting direction of research.

## 1. Introduction

The production of thin-walled products characterized by large sizes requires the use of specific production methods. Due to the possibility of waste-free production of thermoplastic products with relatively low investment costs, rotational molding technology is increasingly used. It displaces the technologies used so far, such as lamination or injection molding [1,2,3]. Rotational molding of thermoplastic polymers is a low-shear technology that, thanks to the long-lasting forming process, allows us to obtain thin-walled ready-to-use products without internal stress in one technological operation. In the process, the feed material in the form of polymer powder is introduced into the mold cavity, which is then subjected to the rotation, usually in two axes, and then heated above the melting point of the polymer material. The slow rotational movement of the mold excludes the occurrence of centrifugal forces. The free coating of unmelted material fractions inside the rotating mold allows us to obtain uniform wall thickness of the product. After the material has completely melted, the mold and the material stacked at the internal surface of the mold are cooled [1,2]. Rotational molding technology can also be considered beneficial from an ecological point of view; the aspect of low material losses and possibility of material recycling of after-use products compared to other thin-walled products manufacturing technologies, including extrusion blow molding or laminating, partly compensates for the significant amount of energy needed to produce a single product [4,5].

Currently, more than 95% of the market of materials intended for shaping in the technology of rotational molding is polyethylene (high-density (HDPE), low-density (LDPE), and linear low-density polyethylene (LLDPE)), the remaining materials are polyamide (PA), poly(vinyl chloride) (PVC), polypropylene (PP) as well as biodegradable polymers, including poly(ε-caprolactone) and the increasingly popular polylactide (PLA). Due to the nature of the technological process, currently, much more emphasis is placed on the development of new types of materials intended for shaping in this technology than the technology and design solutions of machines and devices [6,7]. One of the most dynamically developed research topics is the rotational molding of the polymer composites, in particular those containing filler materials from sustainable sources, including natural lignocellulosic fillers. So far, published examples of rotomoulded composites based on polyethylene (PE) and polylactide (PLA) are based on wood flour or natural fibers [8,9]. The use of natural powder fillers when shaping polymer composites in the technology of rotational casting brings many benefits, such as a significant increase in material rigidity and improvement in the aesthetics of products [10]. However, the tests carried out to date have also shown a significant reduction in the impact strength of composites when more substantial amounts of filler are introduced. The hydrophilic nature of natural fillers causes the risk of insufficient interfacial adhesion, as well as the risk of product porosity due to the release of residual moisture from the filler particles. Therefore, in the presented studies, linear low-density polyethylene (LLDPE) was chosen as a polymeric matrix, as a material with excellent impact resistance in rotational molding applications [11].

Moreover, if the process parameters are not correctly selected, there is a risk of thermal degradation of the organic fraction of the composite. While the use of chemical modification of the filler, such as silanization or mercerization, leads to an increase in the hydrophobicity of lignocellulosic materials [12], in the case of rotational molding it can also lead to deterioration of mechanical properties compared to composites containing unmodified fillers [13]. Much more favorable effects were obtained when using maleated polyethylene (MAPE) to modify lignocellulosic fillers [14].

Another research trend observed for the wood polymer composites (WPCs) is not only intended for rotational molding but is also related to the introduction of new types of fillers. They are often wastes or by-products from various industrial processes, which do not have an industrial application or, despite some, there is still a significant surplus on the material. Among such fillers can be mentioned different types of spent grains [15], husk [16], or brans, for example, wheat bran [17] or natural fibers with an agro-industrial waste origin such as agave fiber [18]. Wheat bran can be considered a by-product during the production of refined grains. Even though the food industry uses bran, it is generated in such quantities that other methods of utilization are also sought.

An excellent example is the use of bran for the production of disposable plates by Biotrem company [19,20,21]. This solution is aimed at partial replacement of plastics. A similar effect can be obtained by using bran as a filler in polymer composites, which was investigated in multiple studies [22,23]. An interesting feature of wheat bran is its composition. Unlike conventionally applied lignocellulosic fillers, such as wood flour, it contains not only fiber but also a significant amount of starch and proteins, which can act as plasticizers of resulting WPCs and enhance their flowability [24,25]. Such an effect would be beneficial for the application of composites containing wheat bran in rotational molding, which requires proper flowability of molten material. In our previous works associated with the introduction of wheat bran into a polymer matrix, we noted the beneficial impact of protein content on the melt flow index of polymer melt [26,27]. Incorporation of wheat bran resulted in the enhancement of flowability compared to olive stone flour, while the highest values of melt flow index were noted for composites containing brewers’ spent grain, a by-product from the brewing industry, which contains over 21 wt.% of proteins. 

Formela et al. [28] showed that wheat bran could be successfully applied as a low-cost alternative for traditional lignocellulosic fillers applied in the manufacturing of WPCs, such as wood flour. The application of wheat bran provided a similar level of mechanical performance. Because of the higher content of amorphous cellulose and the presence of amino acids, it showed a beneficial influence on the processing of natural rubber composites, reducing torque during curing.

In the presented paper, we aimed to investigate the possibility of the application of wheat bran as a filler for linear low-density polyethylene-based composites manufactured by rotational molding. The impact of filler loading on mechanical performance, as well as rheological behavior, fundamental in the manufacturing of thin-walled products via rotational molding, was investigated. 

## 2. Experimental 

### 2.1. Materials

Linear low-density polyethylene type PE 61-011, applied as a matrix in prepared composites, was acquired from Promens (Otwock, Poland). It was characterized by a melt flow index of 6 g/10 min (190 °C, 2.16 kg). 

**Figure 1:**
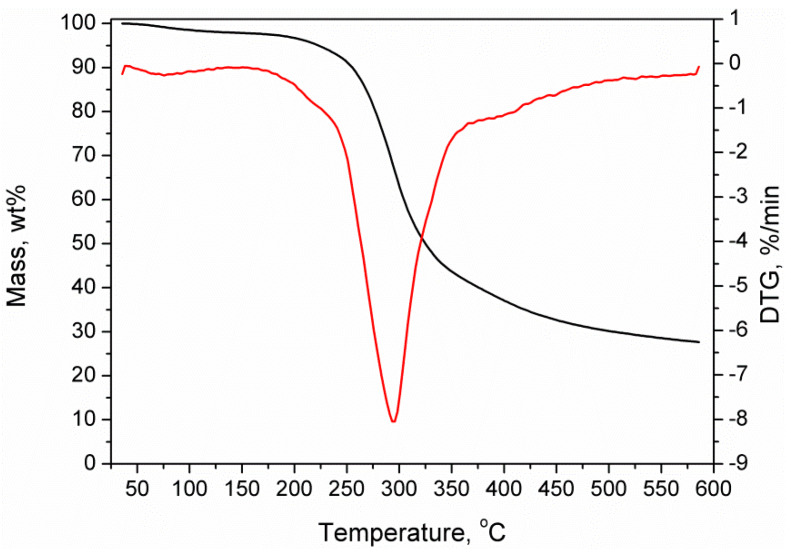
Results of the thermogravimetric analysis of applied wheat bran filler.

Wheat bran was obtained from Młyn Gospodarczy Sp. J. (Pruszcz Gdański, Poland). Before the application in the manufacturing of WPCs, it was ground in co-rotating twin-screw extruder EHP 2 × 20 Sline from Zamak (Skawina, Poland), according to the patent application [29]. The temperature of the extruder barrel was 180 °C, screw speed 200 rpm, and yield of the process 2.4 kg/h. Figure 1 presents the results of the thermogravimetric analysis of pretreated wheat bran filler. It can be seen that it is stable up to 180 °C, except the small mass loss related to the absorption of moisture from the air, associated with the hygroscopic nature of lignocellulosic materials.

**Figure 2:**
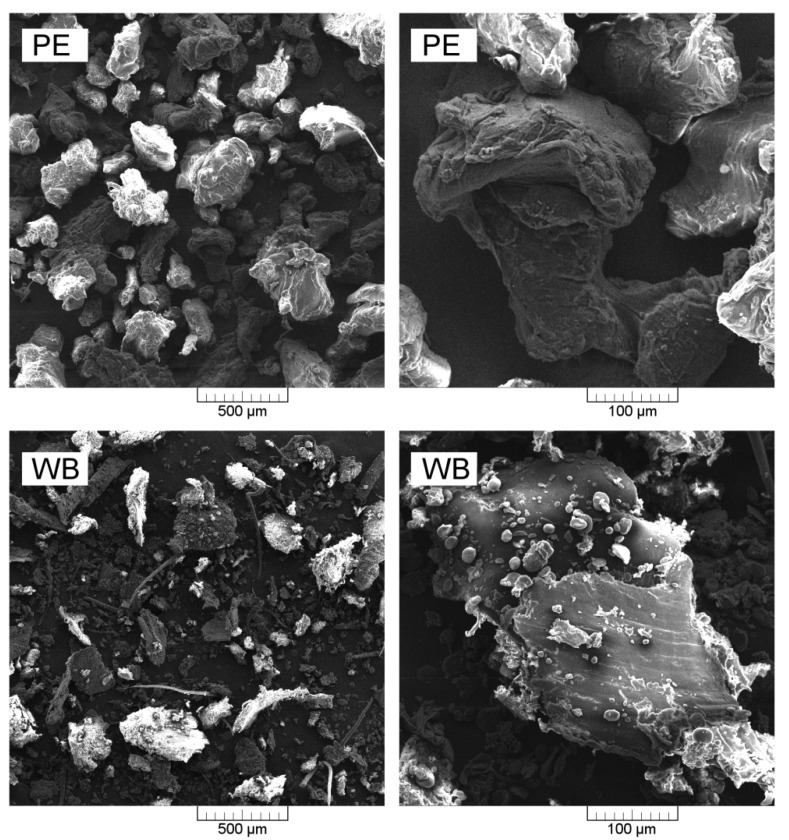
Scanning electron microscope (SEM) images of polyethylene powder and wheat bran.

Applied wheat bran was characterized by a density of 1.272 g/cm^3^. The particle size distribution of the wheat bran (WB) filler is presented in Table 1, as the percentage fractions retained on particular sieves. The chemical composition of applied wheat bran was reported in our previous work [27]. Regarding elemental analysis, WB filler contains almost 45% of carbon, 6.3% of hydrogen and 2.5% of nitrogen, indicating the presence of proteins. Moreover, literature data [30] indicates that WB is composed mainly of hemicellulose (~26%), cellulose (~21%), proteins (~14%), starch (~9%) and lignin (~5%), with varying contents of ashes and lipids. Figure 2 presents scanning electron microscopy (SEM) images of both used materials (PE powder and WB), taken with two different magnifications. It can be seen that polyethylene powder is characterized with comparable particle size, while natural particle-shaped filler varies in its dimensions. Most of the polyethylene particles are in the range between 200 and 500 µm. The biggest particles of WB are comparable with PE, however high amounts of smaller fractions, as well as fillers with high aspect ratio, are observed.

### 2.2. Rotational Molding of Polymer Composites

**Figure 3:**
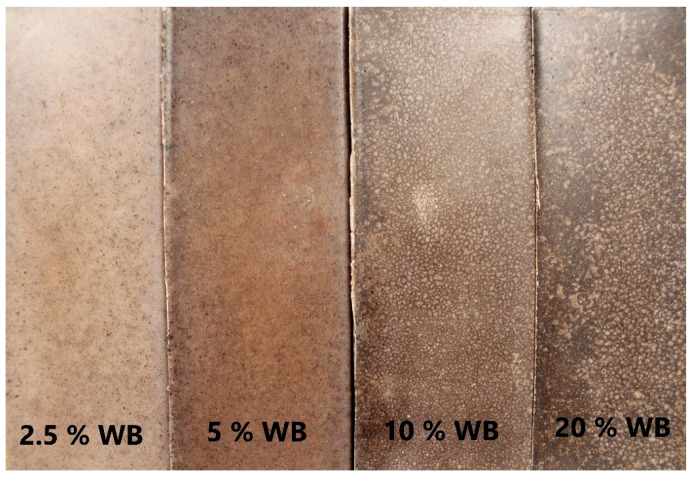
Rotationally molded products polyethylene (PE) with different loadings of wheat bran.

Following the work of Höfler et al. [31], the use of melt blending of composite ingredients was omitted. They found that in the case of the use of linear medium density polyethylene, the difference between preliminary dry blended materials and compounded was negligible. Therefore it can be supposed that a similar effect will be observed for the LLDPE matrix. Before being processed, the polyethylene powder with particle-shaped lignocellulosic filler portions was preliminary mixed with using high-speed knife mill Retsch GM 200 with the rotational speed of 2000 rpm in time of 5 min, and after that subjected to drying process using Memmert ULE 500 laboratory dryer at 70 °C for 24 h. The total amount of material used for the manufacturing of each part was 100 g. The rotational molding process was carried out with using of the single-arm shuttle rotational molding machine REMO GRAF (Poland) with two rotation axes, equipped with a cuboid-shaped steel mold with the cavity dimensions of 185 × 60 × 60 mm. The detailed description of the used machine was presented in our previous work [13]. After introducing a measured amount of polymer and filler, the sealed mold was introduced into the heating chamber of the machine. The following process parameters were used: rotational speed of 1st axis 15 rpm, the rotational speed of 2nd axis 5 rpm, and the set of processing parameters that allows obtaining the constant measured temperature in the oven during the processing of 200 °C, rotation time at elevated temperature 25 min, cooling time using forced air circulation 30 min. Figure 3 shows the rotationally molded products made of PE composites with different filler content. As described in our previous work [13], the applied temperature program was suitable for the manufacturing of composites containing lignocellulosic fillers without risk of their thermal degradation.

### 2.3. Measurements

The chemical structure of wheat bran and composites was determined using Fourier transform infrared spectroscopy (FTIR) analysis performed by a Nicolet Spectrometer IR200 from Thermo Scientific (Waltham, MA, USA). The device had an attenuated total reflectance (ATR) attachment with a diamond crystal. Measurements were performed with 1 cm^−1^ resolution in the range from 4000 to 400 cm^−1^ and 64 scans.

The scanning electron microscope (SEM)—model MIRA3—produced by the Tescan (Brno, Czech Republic), was used in order to assess the structure of the external and internal surface of rotationally molded products. The structures of the surfaces of the rotationally-molded samples were assessed with an accelerating voltage of 1 kV. 

The dynamic mechanical analysis (DMA) was conducted on a DMA Q800 TA Instruments apparatus (TA Instruments, New Castle, DE, USA). Samples with dimensions of 40 × 10 × 2 mm were loaded with variable sinusoidal deformation forces in the single cantilever bending mode at the frequency of 1 Hz under the temperature rising rate of 4 °C/min, ranging the temperature from −120 to 100 °C.

The density of samples was measured based on the Archimedes method, as described in International Organization for Standardization (ISO) 1183. Accordingly, all measurements were carried out at room temperature in methanol medium. 

The tensile strength and elongation at break were estimated following American Society for Testing and Materials (ASTM) D638 standard, using dumbbell samples type IV. Tensile tests were performed on a Zwick/Roell Z020 apparatus (Zwick Roell, Ulm, Germany) with a cell load capacity of 20 kN at a constant speed of 50 mm/min. Each evaluation was prepared for 7 test specimens. 

Shore hardness type D was estimated using Zwick 3131 durometer following ISO 868 (Zwick Roell, Ulm, Germany). 

The rebound resilience was determined with a Schob type pendulum Zwick 5109 (Gibitre Instruments, Bergamo, Italy). The analysis was based on the ISO 4662 standard, originally applied for the evaluation of elastomers’ elasticity. Here it was applied to determine the wheat bran content on the damping properties of composites. Each evaluation was prepared for 7 test specimens.

Rheological investigations were carried using an Anton Paar MCR 301 rotational rheometer (Anton Paar, Ostfildern-Scharnhausen, Germany), with 25 mm diameter parallel plates with a 0.5 mm gap under the oscillatory mode. The experiments were conducted at 190 °C. In order to realize the dynamic oscillatory measurements, the strain sweep experiments had to be proceeded. The strain sweep experiments of all the samples were performed at 190 °C with a constant angular frequency of 10 s^−1^ in the varying strain window 0.01–100%. The strain value, determined during the preliminary investigations and used during the frequency sweep experiments, was set as 0.5% and was located in the linear viscoelastic (LVE) region for all samples. The angular frequency used during studies was in the range of 0.05–500 s^−1^. 

Melt flow index of PE and composites was carried out using Dynisco LMI 4004 plastometer (Dynisco, Franklin, USA), according to ISO 1133. The measurements were conducted at a temperature of 200 °C under a 2.16 kg load.

Vicat softening point temperature (VST) investigations were prepared with the use of the Testlab RV300C apparatus (Testlab, Warszawa, Poland). Measurements were carried out in an oil bath following ISO 306 standards correspondingly. Vicat softening temperature was determined with a load of 10 N and a heating rate of 120 °C/h.

## 3. Results and Discussion

### 3.1. Chemical Structure

**Figure 4:**
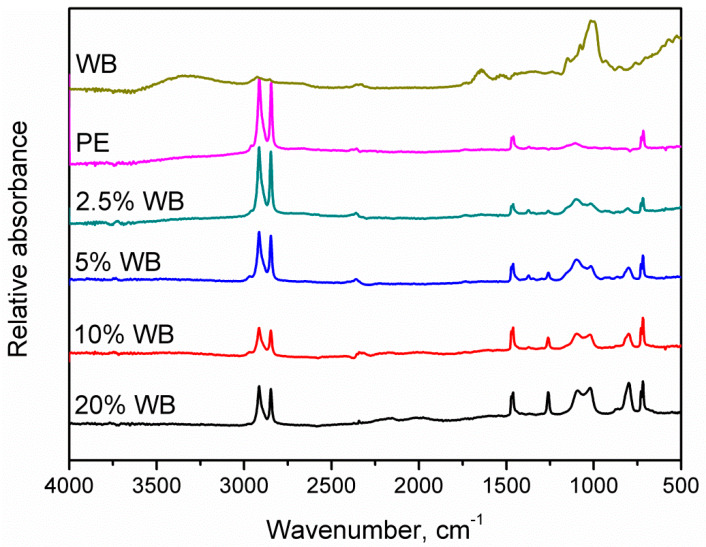
Fourier transform infra-red (FTIR) spectra of prepared composites.

Figure 4 presents the FTIR spectra of prepared composites, as well as raw materials used for their preparation—linear low-density polyethylene and wheat bran. It can be seen that the incorporation of lignocellulosic filler into the LLDPE matrix significantly influences its chemical structure. Spectra plotted for neat matrix and filler show typical appearance for these types of materials, and are in line with data presented by other researchers [32,33]. Signals at 2845 and 2915 cm^−1^ were associated with the symmetric and asymmetric stretching vibrations of C–H bonds, which are present in polyethylene backbone, but also in the structure of natural polymers present in wheat bran. Moreover, signals attributed to the bending, wagging and twisting deformations of these bonds were observed in the ranges of 1457–1468, and 715–726 cm^−1^, respectively. Beneficially, presented FTIR spectra indicate a lack of signals corresponding to carbonyl groups resulting from the degradation of the polyethylene matrix, which is often present around 1740 cm^−1^ [34]. For WB, a broad peak attributed to the presence of hydroxyl groups in cellulose particles was observed around 3330 cm^−1^. Other significant signals observed for introduced filler were noted around 1643 cm^−1^ (stretching vibrations of unconjugated C=O and C=C bonds in polysaccharides), 1530 cm^−1^ (HOC in-plane bending vibrations, characteristic for carbohydrates), 1150 cm^−1^ (stretching of C–O and C=O bonds), 1078, and 996–1015 cm^−1^ (stretching vibrations of C–O bonds) [27]. Incorporation of WB filler into the LLDPE matrix caused the appearance of peaks at 1260, 1095, 1020, and 800 cm^−1^. They were attributed to the stretching vibrations of C–O, C–O–C bonds, introduced by the wheat bran. 

### 3.2. Microscopic Structure

**Figure 5:**
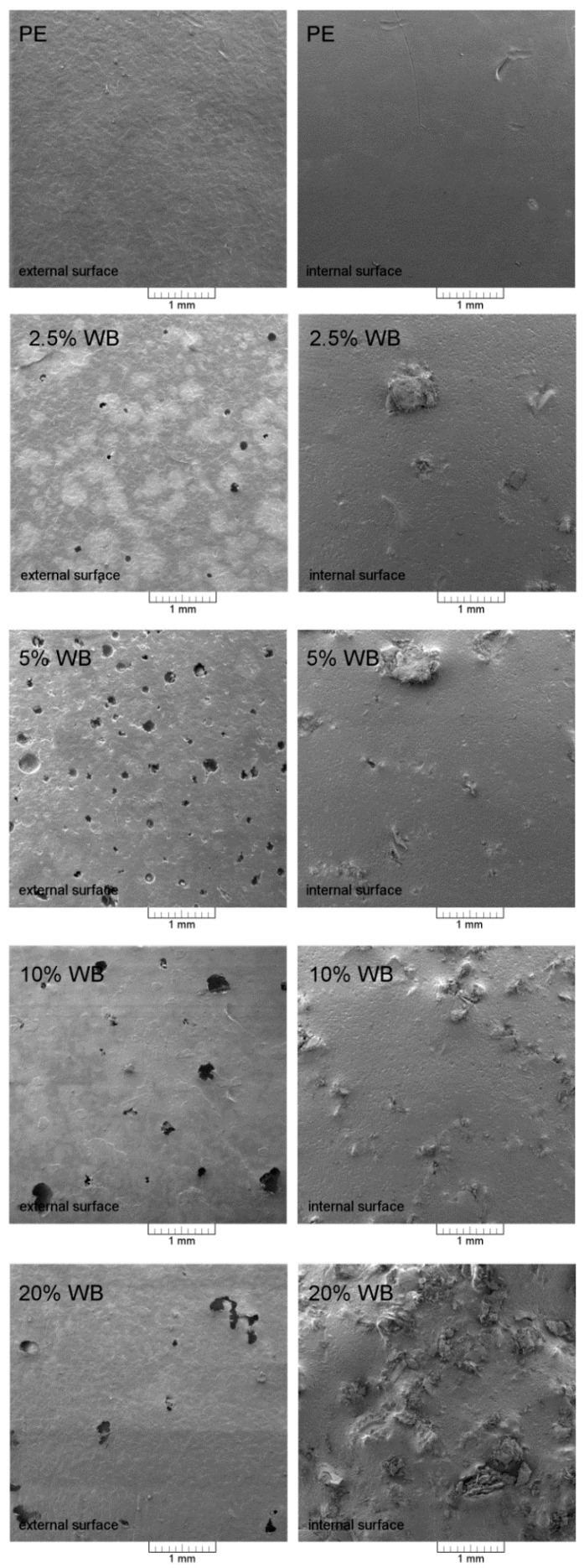
SEM images of rotationally-molded PE and PE-WB composites.

Figure 5 shows SEM images presenting the external and internal surface of the rotationally molded samples made of PE and PE-based composite with various amounts of WB. The absence of exposed filler particles on the outer surface of the products is essential from the application point of view. The polymer layer formed at the surface of the thin-walled product limits the swelling of the organic filler particles dispersed in the matrix. This phenomenon suggests the proper realization of the technological process, the presence of the lignocellulosic particles, did not suppress the first step of the product forming connected with the sintering of the polymer powder at the mold surface. Importantly, it should be emphasized that while in all composite samples, the presence of filler particles is observed on the inner surface, the outer surface of all composites is characterized by a coherent outer coating formed by polyethylene. One of the most significant quality problems of rotationally molded products is the presence of ‘pin-holes’ at the surface [35]. Unfortunately, the addition of even the smallest amount of lignocellulosic filler caused the presence of the mentioned quality failure. In the case of composite samples, defects in the form of pin-holes are observed, the most visible for 5% WB sample. 

**Figure 6:**
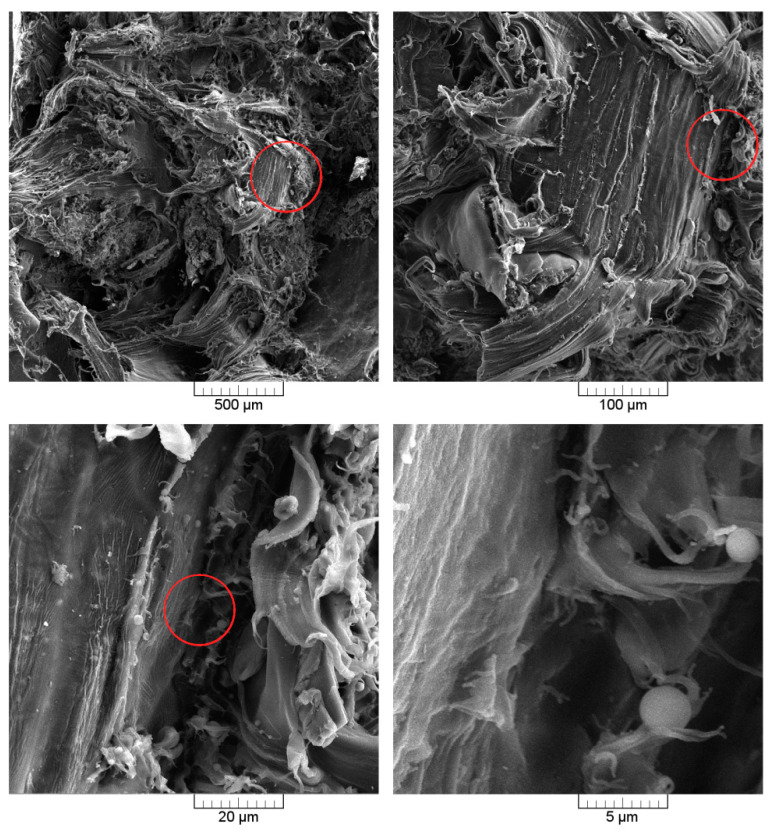
SEM images of 20% WB composite cross-section, taken with different magnifications.

It should be noticed that no loose filler particles not sintered or covered to the polymer matrix were observed inside the product. The observed WB particles at the internal surface of the product, despite the incomplete coverage by the polymer matrix, are embedded in the sintered polyethylene. Moreover, it could be stated that the adhesion at the interphase seems to be at an acceptable level (no gap between the continuous polymer phase and the filler, which is especially visible in the case of a composite containing 20 wt.%). Figure 6 presents SEM images of the cross-section of the composite with 20 wt.% WB content. Four images taken with increasing magnification allow us to observe the interfacial region between the polymer and organic filler. It can be seen that the smallest fractions of wheat bran are loosely connected with the polymeric matrix. The biggest particles, with a size comparable to the size of polyethylene, are well saturated by the polymeric matrix, which can be observed, especially in photographs taken with the highest magnification (Figure 6). Despite the realization of the fracture of 20% WB composites at room temperature, the large filler particles were not pull-out of the polymeric matrix subjected to plastic deformation. Cross-section SEM images confirm, determined by indirect method, the porosity of the composite with the higher filler content. Other research groups prepared rotationally-molded composites based on polyethylene matrix filled with maple and agave fibers, which did not contain proteins [8,10]. In the case of those materials containing 10 wt.% to 30 wt.% of fibers, poor adhesion, and significant voids at the interphase were noted. Therefore, it can be concluded that the presence of proteins in wheat bran, can enhance the interactions of filler with the polymer matrix.

### 3.3. Physico-Mechanical Properties

Mechanical performance of materials prepared by rotational molding strongly depends on the porosity of the materials; therefore, it is important to investigate this issue [36]. Table 2 presents the experimental and theoretical values of materials’ density, which were calculated following the Equation (1):

(1) $$\rho_{C}=\rho_{PE}\cdot \left(1-\varphi_{F}\right)+\rho_{F}\cdot \varphi_{F},$$

where: $\rho_{C}$—density of the composite, g/cm^3^; $\rho_{PE}$—density of the polyethylene, g/cm^3^; $\rho_{F}$—density of the filler, g/cm^3^; $\varphi_{F}$—a fraction of the filler. 

Using these values of the density, the composite’s porosity was calculated according to Formula (2):

(2) $$p=\left(\left(\rho_{theo}-\rho_{exp}\right)/\rho_{theo}\right)\cdot 100\%,$$

where: *p*—porosity of the material, %; $\rho_{theo}$—theoretical value of density, g/cm^3^; and $\rho_{exp}$—the experimental value of density of composite, g/cm^3^.

As mentioned above, porosity is one of the most important factors influencing the mechanical performance of materials obtained by rotational molding. Another essential factor in the case of composites is interfacial adhesion, determining the strength of interfacial interactions, hence compatibility between matrix and filler. Adhesion can be determined and quantitatively described using the concept of adhesion factor, developed by Kubat et al. [37]. It is based on the fact that tanδ of the composite material consists of several components attributed to the matrix, filler, and interphase, and can be described with the following mathematical Formula (3):

(3) $$\tan\delta_{C}=\varphi_{F}\cdot \tan\delta_{F}+\varphi_{I}\cdot \tan\delta_{I}+\varphi_{PE}\cdot \tan\delta_{PE},$$

where: *C*, *F*, *I,* and *PE* subscripts are related to composite, filler, interphase, and polyethylene, respectively, while φ stands for the volume fraction of each phase. 

Comparing to polymer matrices, fillers are usually significantly more rigid, so for simplification, it can be assumed that the damping of the filler is very low. Therefore its influence on the adhesion factor can be neglected. Hence, Formula (3) may be rearranged into (4):

(4) $$\left(\tan\delta_{C}/\tan\delta_{M}\right)≅\left(1-\varphi_{F}\right)\cdot \left(1+A\right),$$

where adhesion factor (*A*) is described by the following Formula (5):

(5) $$A=\left(\varphi_{I}/\left(1-\varphi_{F}\right)\right)\cdot \left(\tan\delta_{I}/\tan\delta_{PE}\right)-1.$$

Comparing to the share of matrix and filler, the volume fraction of interphase is very low. Moreover, the aspect ratio of applied filler is rather low. Hence transcrystallinity of the interphase may be neglected, and damping of interphase may be considered similar to the damping of the matrix. Therefore, Formula (5) may be rewritten to express the adhesion factor in terms of the relative damping of composite and matrix, and the volume fraction of filler. Such rearrangement leads to (6):

(6) $$A=\left(1/\left(1-\varphi_{F}\right)\right)\cdot \left(\tan\delta_{C}/\tan\delta_{PE}\right)-1.$$

**Figure 7:**
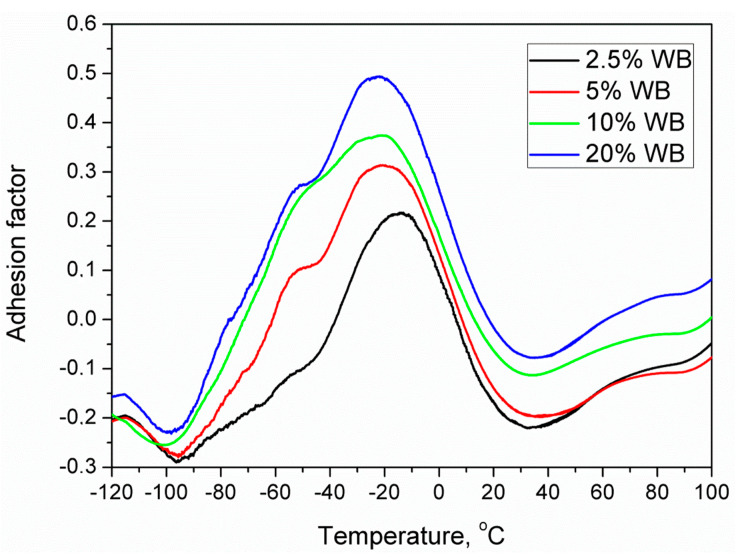
Adhesion factor vs. temperature of rotationally molded PE and PE-WB composites.

Because the value of the adhesion factor depends on the loss tangent of a particular material, it is temperature-sensitive. Therefore, in Figure 7, there are presented temperature plots of adhesion factor for investigated materials. Values of tanδ were obtained using dynamic mechanical analysis and were recalculated using a set of equations mentioned above. 

Considering above mentioned formulas, lower values of A factor are indicating higher interfacial adhesion inside the composite. An increase of A is associated with higher damping ability of composite, caused by the “loss” of the energy inside the material when stress is applied. Such “loss” is related to the viscoelastic nature of polymer matrix, but also the dissipation of energy by various motions inside the composite. These motions are favored by the porosity of the material, and by insufficient interfacial adhesion, which enables sliding of macromolecular chains on the surface of filler particles. Such an effect can also be enhanced by the presence of proteins in wheat bran, which was confirmed in our previous works [26,27].

**Figure 8:**
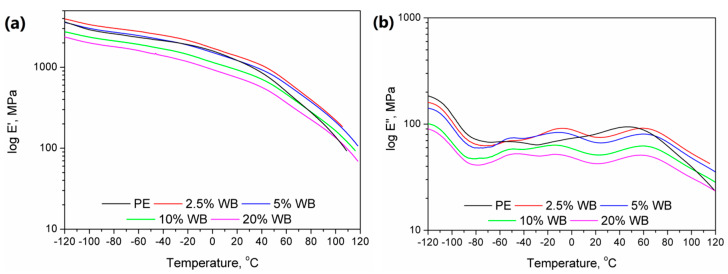
Temperature dependence of (**a**) storage (E’) and (**b**) loss (E”) modulus of prepared materials.

Moreover, except for the values of the adhesion factor calculated from tanδ, in Figure 8, there are also plotted temperature dependence of storage and loss modulus of prepared materials. It can be seen that the values are relatively similar for all analyzed composites. Nevertheless, an interesting decrease of the storage modulus with the addition of wheat bran was noted, which is not typical for the incorporation of fillers into the polymer matrix. Such an effect may be associated with the increase of porosity and the increase of adhesion factor, indicating deterioration of interphase quality. Moreover, some changes in the particular transitions of the PE phase were noted. Typically, for polyethylene, signals related to the α-relaxation are observed between 20 and even 100 °C, depending on the type and density of PE [38]. This transition is generally affected by the deformation of the interfacial regions, rather than bulk phase. Therefore, the shift of peak associated with the α-relaxation towards lower temperatures and decrease of its magnitude indicates the “looser” structure of prepared samples, confirming the values of the adhesion factor. Popli et al. [39] suggested similar dependences associated with the b-relaxation occurring in the presented case around −10 °C.

It can be seen that the increasing content of wheat bran in analyzed composites caused the increase of the adhesion factor. Among other factors, such an effect was related to the increasing porosity of the structure, which significantly affected the mechanical performance of the material. Moreover, due to the hydrophilic nature of lignocellulosic fillers and hydrophobic character of polyethylene, interfacial interactions between matrix and filler are rather weak, which could be overcome by proper modification of filler before compounding [40].

Negative values of adhesion factor observed on the plot are due to the simplification of calculations related to neglecting of the filler anisotropy and with development of the unconsidered interphase region, which have a slight influence on the macromolecular mobility at the filler surroundings [41].

**Figure 9:**
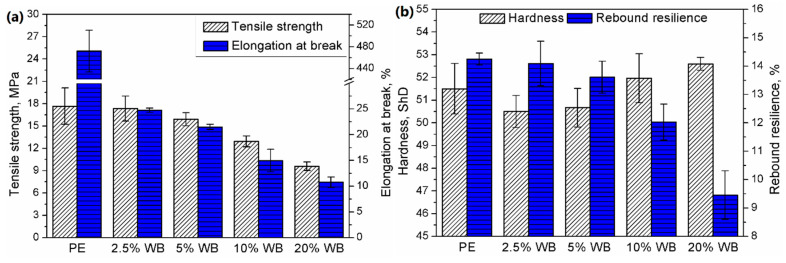
Mechanical properties: (**a**) tensile strength and elongation at break, and (**b**) hardness and rebound resilience of rotationally molded PE and PE-WB composites.

**Figure 10:**
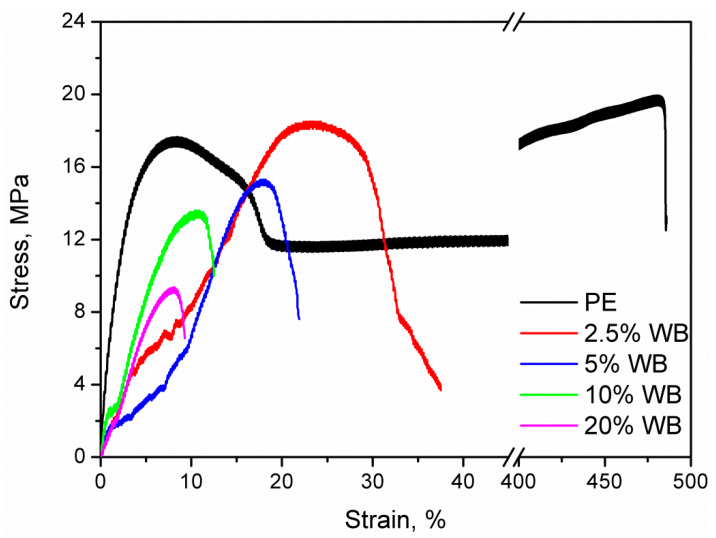
Exemplary stress-strain curves of prepared materials.

**Figure 11:**
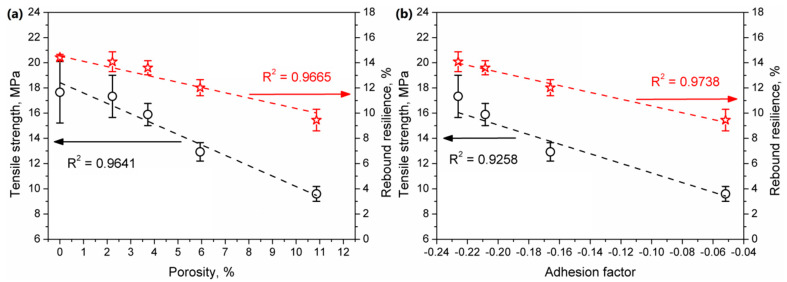
The impact of (**a**) porosity and (**b**) adhesion factor on the tensile strength and rebound resilience of the rotationally molded samples.

Figure 9 presents the values of basic mechanical parameters of prepared samples. Moreover, in Figure 10, there are also presented exemplary stress-strain curves for prepared composites. It can be seen that the incorporation of wheat bran into the PE matrix resulted in a noticeable decrease in tensile properties and rebound resilience. Such effect is directly associated with the increase of composites’ porosity and insufficient strength of interfacial interactions, expressed by the adhesion factor. Porosity caused discontinuity of structure, which is weakening the material and results in its lower strength [42]. The presence of proteins may cause sliding of polymer on the surface of filler particles, without the proper stress transfer. Moreover, the presence of pores inside the structure decreases the actual cross-section area of material, which results in lower values of tensile strength obtained during mechanical testing. Dependences of tensile strength and rebound resilience on porosity and adhesion factor are presented in Figure 11. Usually, the incorporation of lignocellulosic fillers into the PE matrix results in the increase of composite hardness [26]. In the considered case, the measured values of the hardness are at a comparable level. The initial decrease of this parameter for lower loadings of filler was related to the presence of free volumes resulting from the increase of porosity. Despite definitely higher porosity of composites containing 10 wt.% and more of WB, slightly higher values of hardness are probably an effect of compensation of the negative porous structure by the presence of rigid WB particles [43].

### 3.4. Rheological Behavior

**Figure 12:**
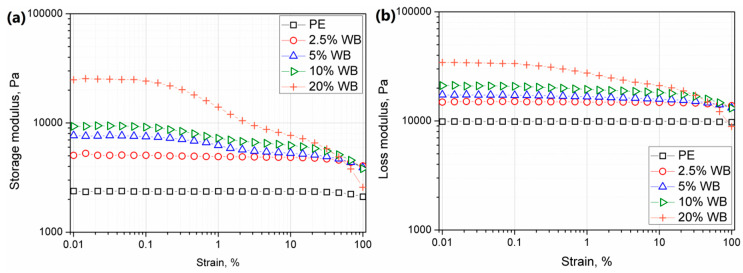
Storage (**a**) and loss (**b**) modulus changes obtained during strain-sweep rheological experiments.

According to Chaudhary et al. [44], the increased melt viscosity of rotationally molded material at low shear rates as well at zero shear viscosity is connected with extending the sintering process and difficult bubble removal during rotational molding. Therefore, the realization of detailed studies using oscillatory rheometrical analysis at low angular frequencies became essential to understand the changes in final product structure and resulting properties changes. Figure 12 shows storage (G’) and loss (G”) modulus changes as a function of strain obtained during strain sweep experiments. Incorporation of lignocellulosic filler caused the increase of both moduli as well as shortening of the linear viscoelastic region. Nevertheless, the increase of modulus was not so substantial, as for the incorporation of wood flour, which can be associated with the enhanced flowability of material due to protein content in wheat bran [16]. While for the pure and composite samples containing up to 2.5 wt.% of the filler LVE range was comparable, further increasing content of the WB caused the effect of a two-step decrease of the storage modulus. According to the literature, an additional plateau observed for samples containing lignocelluloses filler is related to the disruption of initial agglomerated structures of the filler in the molten polymeric matrix [45]. The higher the content of the filler, the shorter the LVE region. The lowered linear region observed for composites containing the highest filler content may be interpreted as decreased interparticle bonding physical forces of the filer hindered network at lower strain values [46,47]. It should be noticed that for all tested materials G” values were higher than G’, so despite additional rheological effects occurred at low strain values, the low viscous behavior dominates. 

**Figure 13:**
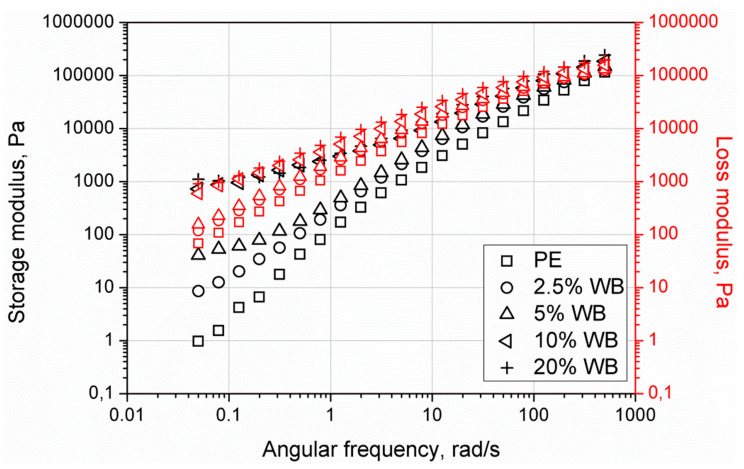
Storage and loss modulus vs. angular frequency curves of rotationally molded PE and PE-WB composites.

**Figure 14:**
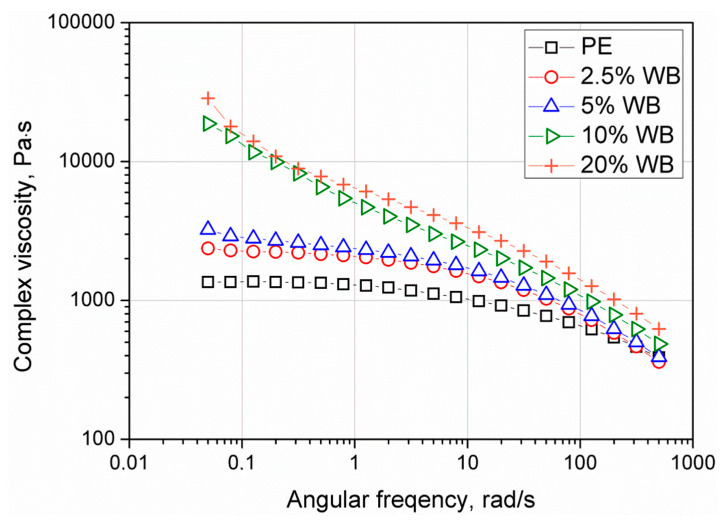
Complex viscosity curved of rotationally molded PE and PE-WB composites.

The results of frequency sweep experiments realized in the LVE region are presented as changes of G’, G” (Figure 13), and complex viscosity |η*| (Figure 14) as a function of angular frequency. Composites with wheat bran content up to 10 wt.% showed a viscous like response in the applied angular frequency (ω) range. Only for composite filled with 20 wt.% of WB the higher values of G’ were observed at low and high ω values. The solid-like viscoelastic behavior results from the previously mentioned formation of filler percolation network [48]. Moreover, with the increasing content of the filler, higher values of moduli were observed. While a sample containing 2.5 wt.% of WB showed the terminal slope of the G’ curve as unfilled PE, the higher amount of the filler, that is, 5 wt.% and more, showed the change in the slope of G’(ω) curves at low angular frequencies. As a result, the creation of lowered dependency of G’ by ω at low, in case of 10 wt.% and 20 wt.% of the filler, transform the curve shape into the plateau. This phenomenon may be attributed to the creation of large agglomerated structures of the filler. Complex viscosity curves presented in Figure 14 showed shear-thinning behavior for all composites. However, the Newtonian flow region was observed for materials containing 2.5 wt.% and 5 wt.% of WB. Higher amounts of filler caused the decay of this phenomenon, which is directly related to the interlocking of the filler particles [49,50]. Although all of the materials showed limited flow behavior at low angular frequencies corresponding to those processing conditions during rotational molding, the dominating viscous behavior suggests that all composite materials are still suitable for processing. However, a significant increase of the viscosity at low angular frequencies, as well as inability to a proper determination of zero-shear viscosity by fitting experimental data to Carreau-Yasuda model for composite samples, may be related to increased porosity measured for composites (exceptionally distinct for 10 wt.% and 20 wt.% of the WB) [35].

The materials used for shaping products using the rotational molding method are mainly graded based on density and rheological properties described by MFI. Both parameters are significantly connected with both the performance and the quality of final products. The use of materials with high MFI values llows the shaping of complicated shape products and composites, in which the addition of fillers increases the viscosity of the composition [51]. The different materials may show the comparable MFI, which is often used as the criterion of the choice of the polymeric grade for rotational molding, however, due to different molecular weight distributions, they may vary with the zero-shear viscosity which reflects the beginning of the forming process (melting) and may be connected with the creation of structural defects [52]. The MFI values measured for PE and PE-WB composites are shown in Table 3. It can be seen that even the lowest filler content (2.5 wt.%) caused more than a 30% reduction in MFI value compared to unmodified PE. Further increase in the filler content resulted in a further reduction of the MFI value, up to a value of 3.11 g/10 min for 20 wt.% of the filler. A similar effect of MFI reduction was observed for high-density polyethylene filled with wood particles [53]. Obtained results suggest the worsened processability of the composite materials in comparison to pure PE. It should be remembered that while MFI values may be useful for preliminary selection and characterization of the materials suitable for the rotational molding process, the values of the MFI are determined in different shearing conditions to those during preliminary sintering and further forming [35,54].

### 3.5. Thermomechanical Behavior

**Figure 15:**
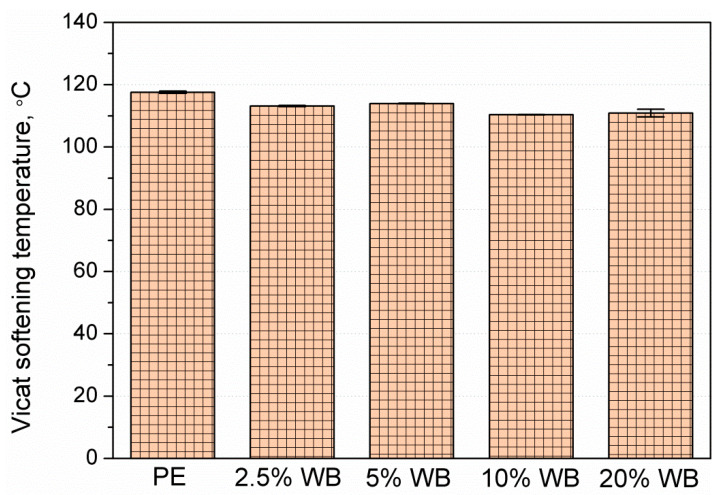
Vicat softening temperature values of rotationally molded PE and PE-WB composites.

The results of the heat resistance measurements taken for PE-WB composites revealed minimal changes (Figure 15). The initial VST for pure PE (117 °C) was the highest, while the heat resistance for the increasing WB content was lower, with the addition of 20% WB it was around 111 °C. Such a scale of changes can be considered small, but interestingly the addition of a filler causes thermal resistance folds. Previous research on the rotational molding technique partly overlaps with the obtained VST measurement results, where the addition of the filler did not significantly affect the heat resistance of the composite [55,56], or even worsened it [57]. Usually, the occurrence of significant material porosity is indicated as the main reason for the deterioration of materials properties of rotomolded composites [58].

## 4. Conclusions

The presented research paper aimed to investigate the impact of wheat bran on the structure, physico-mechanical, rheological, and thermo-mechanical performance of rotationally-molded composites based on an LLDPE matrix. Research associated with the manufacturing of wood polymer composites using this processing method are still somewhat limited, but it is an exciting direction of research from both economic and ecological points of view. Moreover, wheat bran, due to the content of proteins, also provides other qualities (finer interphase and lower stiffness) to the polymer composites compared to conventionally applied lignocellulosic fillers such as wood flour. Rheological tests did not indicate significant disturbances in the flowability and processing of composite polymer melts, which could be associated with the presence of proteins in wheat bran. Higher values of moduli were noted, which was associated with the incorporation of solid particles. Moreover, shear-thinning behavior was observed, which makes prepared materials suitable for rotational molding. Such an effect is very beneficial for their potential application. As a result of satisfactory rheological properties and flowability of composites, the microscopic analysis indicated the acceptable quality of the interphase with relatively low content of voids and the fine structure. Introduction of 2.5 wt.% and 5 wt.% of wheat bran allowed to maintain the mechanical properties at a similar level, despite the rise of porosity. Higher loadings resulted in the deterioration of mechanical performance, which was also expressed by the noticeable rise of the adhesion factor. Moreover, hardly any changes in thermomechanical stability were noted, which was kept at a similar level.

Generally, the presented results indicate that the manufacturing of thin-walled products based on wood polymer composites via rotational molding should be considered a very interesting direction of research. Future trends in this area should include modifications of filler or matrix aimed at strengthening of interfacial adhesion, which would enhance the mechanical performance and allow to introduce higher amounts of filler, simultaneously reducing the cost of final products.

## Figures and Tables

**Table 1:** Particle size distribution of applied wheat bran (WB) filler.

**Sieve Size, mm**	1.020	0.750	0.500	0.250	0.125	0.063	Residue
**Content, wt.%**	6.68	7.52	2.78	28.71	38.11	16.13	0.08

**Table 2:** Physical and structural properties of PE-WB composites.

Sample	Theoretical Density, g/cm^3^	Experimental Density, g/cm^3^	Porosity, %
PE	0.931	0.931	0.00
2.5% WB	0.951	0.930	2.22
5% WB	0.959	0.924	3.72
10% WB	0.976	0.918	5.93
20% WB	1.009	0.899	10.86

**Table 3:** Melt flow index (MFI) of PE-WB composites.

Sample	Melt Flow Index, g/10 min
PE	6.51 ± 0.19
2.5% WB	4.43 ± 0.07
5% WB	4.14 ± 0.12
10% WB	3.89 ± 0.07
20% WB	3.11 ± 0.05
